# Microbiological Profile and Drug Resistance Analysis of Postoperative Infections following Orthopedic Surgery: A 5-Year Retrospective Review

**DOI:** 10.1155/2022/7648014

**Published:** 2022-07-04

**Authors:** Zuhdi O. Elifranji, Bassem Haddad, Anas Salameh, Shehadeh Alzubaidi, Noor Yousef, Mohammad Al Nawaiseh, Ahmad Alkhatib, Razan Aburumman, Abdulrahman M. Karam, Muayad I. Azzam, Mohammad A. Alshrouf

**Affiliations:** ^1^Department of Special Surgery, Division of Orthopaedics, School of Medicine, The University of Jordan, Amman, Jordan; ^2^The School of Medicine, The University of Jordan, Amman, Jordan

## Abstract

**Background:**

The distribution of postoperative orthopedic infection and their susceptibility pattern to antibiotics vary regionally and change over time. The incidence of methicillin-resistant *Staphylococcus aureus* infection is rising worldwide. Therefore, knowledge of the frequency of the causative microorganisms and their susceptibility to antibiotics are necessary for an improved therapeutic outcome. This study aims to study the frequency and distribution of postoperative orthopedic infection and their resistance pattern to antibiotics.

**Methods:**

The study utilized a retrospective design that took place over a period of 5 years from 2016 and 2020 at a tertiary care hospital. The bacterial culture testing was performed by a recommended method. Descriptive statistics were used to analyze the data.

**Results:**

A total of 158 patients (100 males and 58 females) with positive cultures of postoperative orthopedic infection were included. The most common infective organism was *Staphylococcus aureus*, 64 patients (38.1%); coagulase-negative staphylococci, 40 patients (23.8%); *Klebsiella* species, 14 patients (8.3%); and *Enterococcus* species, *Escherichia coli*, and *Pseudomonas aeruginosa* in 10 patients (6%). Data also showed that gram-positive bacteria were detected in 118 patients (70.8%), while gram-negative microorganisms were found in 50 patients (29.8%). Among *Staphylococcus aureus*, 79.7% were MRSA, and vancomycin was the most effective antibiotic in staphylococcus infections. The antibiotics with the greatest sensitivity to gram-positive bacteria were vancomycin, linezolid, tigecycline, moxifloxacin, and nitrofurantoin, while the antibiotics for gram-negative bacteria with greater sensitivity were tigecycline, amikacin, ertapenem, imipenem, and cefotaxime.

**Conclusion:**

*Staphylococcus aureus* is the most common postoperative orthopedic infection, which was predominantly MRSA with vancomycin being the most effective antibiotic. In addition, the results showed a high resistance pattern to the commonly used antibiotics, leaving few choices. Antibiotic agents should be carefully selected according to specific drug sensitivity through routine monitoring of drug resistance patterns and to help formulate hospital antibiotic policy.

## 1. Introduction

Resistance of bacterial pathogens to commonly used antibiotics and the emergence of multidrug-resistant bacteria are a worldwide challenge that is increasing at an alarming rate, which has led antibiotic choices to become both limited and expensive [1]. Despite numerous actions taken to tackle antibiotic resistance, global trends show no signs of slowing down [2]. As a result, infections with these resistant bacteria will continue to lead to more serious illnesses, treatment failures, prolonged hospital admissions, and an increase in healthcare cost [3, 4]. In the U.S. alone, antibiotic-resistant bacteria cause at least 2 million infections and 23,000 deaths a year and result in an annual economic burden of 55–70 billion dollars [5]. However, in Europe, it is estimated that antimicrobial resistance is correlated with more than nine billion euros per year [4, 6].

In orthopedics, postoperative infections are considered catastrophic complications that significantly increase medical expenses, prolong hospitalization, and can bring about the loss of the operated limb or even death [7, 8]. During these surgeries, implantation materials are used, which increase the risk of infections that can be notoriously hard to eradicate due to biofilm formation. *Staphylococcus aureus* (*S*. *aureus*) is identified as the most common organism causing orthopedic infections, with methicillin resistance rates as high as 63% and around 25% resistant to vancomycin, followed by other pathogens like *Pseudomonas aeruginosa*, *E*. *coli*, and Enterobacteriaceae [7–10]. Furthermore, multidrug-resistant organisms were found in 37.5%–65.5% of postoperative orthopedic infections [11, 12].

In light of the challenge, antimicrobial resistance poses a challenge to healthcare systems worldwide, which is linked with the consequences and burden of postoperative orthopedic infections. This study aimed to find the rate and pattern of postoperative orthopedic infections in a tertiary care hospital to improve the infection control practices and understand the microbial causes and antibiotic resistance patterns among them.

## 2. Materials and Methods

### 2.1. Study Design

The study utilized a cross-sectional retrospective design that took place over a period of 5 years from 2016 and 2020 at Jordan University Hospital (JUH), a tertiary care teaching hospital. The study's objective was to isolate and characterize pathogens in orthopedic patients presenting with postoperative infections and determine their antimicrobial susceptibility pattern. Ethical approval was granted by the Institutional Review Board (IRB) of JUH.

### 2.2. Study Population

The study included all patients that presented to the orthopedic department with bacterial postoperative infection. Patients were considered to have a postoperative infection if the microbial contamination was found within 30 days of operation or one year or if a prosthetic implant was placed [13]. Patients with open fractures were excluded from the study.

### 2.3. Data Collection Process

Clinical data were collected retrospectively using JUH's electronic information system. This included age, gender, date of admission, location of the primary surgery (JUH vs. referred cases from other hospitals), surgery details (name, duration, and whether the surgery is trauma, elective, or emergency), and patients' comorbidities. Laboratory information including the isolated organisms and their antibiotic susceptibility patterns was collected. The following procedure for obtaining the sample, culturing, bacterial identification, and susceptibility testing is used at JUH.

### 2.4. Obtaining the Sample and Culture

Cultures were requested when a postoperative infection was clinically suspected (symptoms of localized tenderness, localized pain, stiffness, erythema, swelling, fever, chills, or rigors, purulent drainage from the superficial incision, leukocytosis on blood tests, and elevated inflammatory markers). The cultures were collected from the surgical site or near it using sterile swabs (if superficial), aspirate, or pus using sterile containers. The specimens were then transported to the microbiology laboratory immediately and processed. The swabs were cultured on 5% blood agar and MacConkey agar and incubated aerobically at 35–37°C for 24 hours. Calibrated loops were used for the semiquantitative method. The isolates were identified and confirmed using a variety of standard techniques, including gram staining, colony morphology, growth on selective media, lactose and mannitol fermentation, H2S production, catalase, oxidase, coagulase, indole, citrate utilization, and urease test. If there was any visible growth, it was identified by standard phenotypic methods and was subjected to antibiotic susceptibility testing.

### 2.5. Antimicrobial Susceptibility Testing

**Figure 1:**
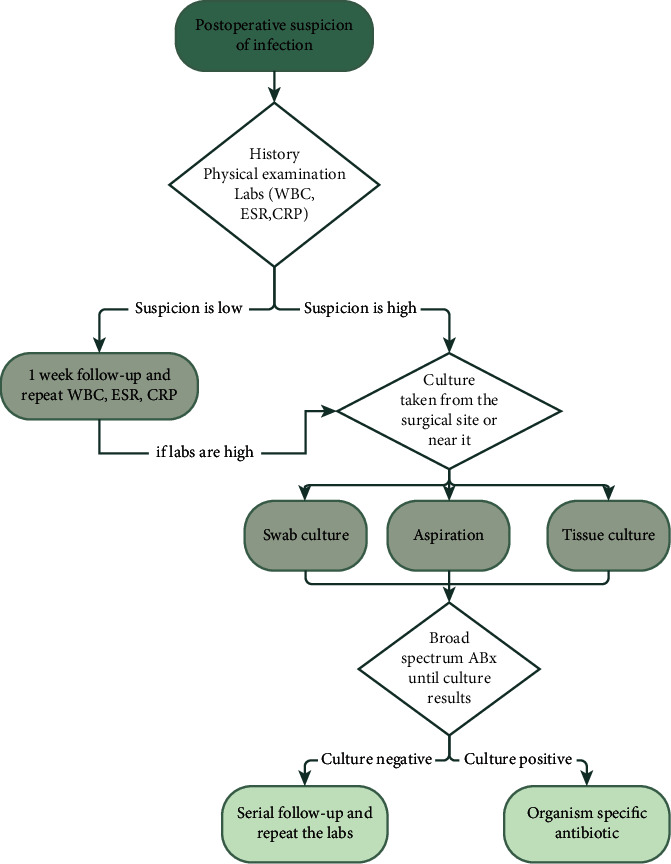
Management protocol in patients with suspicion of postoperative infection.

The antimicrobial susceptibility test was carried out by the Kirby–Bauer disc diffusion method as per the Clinical Laboratory Standards Institute (CLSI) guidelines on Mueller–Hinton agar [14, 15]. Antibiotic discs were placed after 15 minutes of inoculation to Mueller–Hinton agar seeded with each isolate and were incubated for 24 hours at 37°C. The antibiotic discs consisted of many types of antibiotics depending on the type of bacteria, ampicillin, levofloxacin, gentamicin, ciprofloxacin, vancomycin, oxacillin, erythromycin, clindamycin, linezolid, tigecycline, cefotaxime, co-trimoxazole, nitrofurantoin, benzylpenicillin, moxifloxacin, tetracycline, chloramphenicol, rifampin, quinupristin, amoxicillin/clavulanic acid, and cefoxitin. The diameter of the zone of inhibition around the disc was measured using a sliding metal caliper. Cefuroxime 1.5 g IV was administered as a prophylaxis antibiotic in all types of surgeries (elective, trauma, or emergency), while vancomycin 1 g IV was used in the case of known patient allergy.  Figure 1 demonstrates the management protocol in patients with suspicion of postoperative infection.

### 2.6. Statistical Analysis

All data were entered into Microsoft Excel software (version 2016; Microsoft, Redmond, WA), where they were cleaned, polished, and analyzed. Continuous variables were described as the mean (± standard deviation). We used the count (frequency) to describe other nominal variables.

## 3. Results

### 3.1. Demographics

A total of 158 patients who had postoperative orthopedic-related infections from 2016 to 2020 were included in the analysis. Among the participants, the age ranged from 2 to 89 years, with a mean of 44.9 ± 24.79 years. The patients' gender was distributed as 63.3% males and 36.7% females. The most common comorbidity was hypertension (26.4%). The demographic data and various factors associated with postoperative orthopedic-related infections are summarized in Table 1.

In this study, we categorized cases into two groups based on the source of infection, in order to better understand the causes of infection and to better track antibiotic resistance rates. One hundred fifty-eight patients had postoperative surgical infections, most (*n* = 84) after a trauma surgery. Moreover, the most commonly infected site was the hip (*n* = 23), the knee (*n* = 21), the femur (*n* = 21), the pelvis (*n* = 9), and the elbow and the hand (*n* = 8).

Among the postoperative infection in JUH, the incidence of postoperative infection was 1.13%. The average operating time was 126.3 ± 76.4 minutes (range, 15–480 minutes), with more than half (57.1%) of the operations being more than or equal to two hours.

### 3.2. Identified Bacteria

In the vast majority of the cases, we identified one pathogen, but there were eight cases in which two pathogens were identified and only one case in which three pathogens were identified. There were three combinations of bacteria in cultures with mixed infections: three cases with mixed gram-positive and gram-negative bacteria, three all gram-positive bacteria, and three all gram-negative bacteria.

Antibiogram reports showed that more than two-thirds of the isolates were gram-positive 118 (70.2%), and only in 50 (29.8%) cases, it was gram-negative. The most common infective organism was *Staphylococcus aureus* in 64 (38.1%), including methicillin-resistant *Staphylococcus aureus* (MRSA) in 51 patients (30.4%), coagulase-negative staphylococci species in 40 (23.8%), and *Klebsiella* species in 14 (8.3%) (Table 2).

### 3.3. Antibiotic Susceptibility Pattern

The antibiotics with the greatest sensitivity to gram-positive bacteria were vancomycin, linezolid, tigecycline, moxifloxacin, and nitrofurantoin while the antibiotics for gram-negative bacteria with greater sensitivity were tigecycline, amikacin, ertapenem, imipenem, and cefotaxime. Tables 3 and 4 demonstrate the resistance pattern of bacteria to antibiotic agents.

All gram-positive bacteria were susceptible to vancomycin except in one patient (vancomycin-resistant *Staphylococcus aureus*), and 100% of *S*. *aureus* were sensitive to linezolid, nitrofurantoin, and quinupristin. The majority (79.7%) of *S*. *aureus* were MRSA, with a very high degree of resistance observed toward erythromycin, oxacillin, ampicillin, benzylpenicillin, and amoxicillin/clavulanic acid. Moreover, when comparing antibiotic resistance patterns based on the source of infection and location of the surgery, it was noticed that postoperative infection cases referred to JUH have a higher rate of resistance (Table 5).

## 4. Discussion

This study aimed to investigate the incidence, bacterial etiology, and antibiotic susceptibility patterns of the postoperative orthopedic-related infection incidence at the JUH between 2016 and 2020. Our study showed a low infection rate following orthopedic procedures, with the hip being the most commonly affected site. *Staphylococcus aureus* was the most isolated specimen with alarming MRSA predominance, followed by coagulase-negative staphylococci and *Klebsiella* species. Furthermore, gram-positive bacteria showed high resistance to ampicillin, oxacillin, erythromycin, tetracycline, and clindamycin. In addition, gram-negative bacteria were mainly resistant to ceftriaxone, ceftazidime, cefepime, co-trimoxazole, and ampicillin.

The incidence of orthopedic-related postoperative infections in the literature ranges from 2.5% to 41.9% [11, 16–23]. Comparable rates were found in developing countries, such that in a study conducted on 3096 orthopedic patients at a university hospital in Saudi Arabia, the incidence was found to be 2.55% [24]. A higher rate was found in another study conducted at a tertiary hospital in Oman to evaluate the surgical site infection following different orthopedic procedures showing a rate of 8.57% [11]. However, in our study, the incidence was found to be 1.13%. We hypothesize that the reported rate is lower than in other studies due to underreporting and loss to follow-up. In Jordan, there is no effective postoperative follow-up system, so if a patient decides to follow up outside of JUH, the lack of a national-computerized patient record system that links healthcare facilities will prevent JUH from being informed. This is supported by the fact that in a Jordanian prospective study conducted by Hamdan et al. where patients were followed up by the research team, the incidence was found to be 2.8% [18]. Therefore, a national medical record database is needed, which will be used as a tool to standardize the care in Jordan.

Arab countries face a significant MRSA burden, as MRSA rates among *S*. *aureus* infections range from 9% to 69%, with Jordan having one of the highest rates (37%) [24]. Based on our results, MRSA was the most common infective organism, accounting alone for 30.4% of cases. This finding is similar to that reported by Latha et al. where MRSA was the most frequently identified pathogen in 27.7% of patients [9]. The high rate of MRSA among our patients raises concerns about the emergence of antibiotic resistance in Jordan, especially when considering the inappropriate dispensing of antibiotics and the poor knowledge of the population regarding antibiotic resistance [25, 26].

Antibiotic resistance is a major global issue that has led to 2.8 million antibiotic-resistant infections and 35000 deaths in the United States each year. The rapid emergence of these strains led the Centers for Disease Control and Prevention (CDC) to utilize a list used as a reference in order to detect these pathogens and take appropriate actions [27]. Regarding *S*. *aureus*, our results showed high resistance to oxacillin, ampicillin, benzylpenicillin, and amoxicillin/clavulanic acid 77.4%, 91.7%, 96.9%, and 100%, respectively. Xie et al. reported that *S*. *aureus* had high resistance to penicillin and ampicillin 97.89% and 100%, respectively, which is in line with our findings [28]. However, the oxacillin resistance rate in our study is higher than that of other studies. Hassan et al. found a rate of 53.3%, and in another study, it was 52.4% [28, 29]. In the light of these findings, an effective intervention must be implemented as the high resistance rates to those common antibiotics will be reflected in the treatment efficacy sooner or later. Although the usage of broad-spectrum antibiotics is restricted to prevent further emergence of resistant strains, our results revealed that *S*. *aureus* had higher rates of vancomycin and tigecycline-resistant strains when compared to the global prevalence [30, 31]. These striking findings raise questions regarding overprescription of broad-spectrum antibiotics in Jordan.

**Figure 2:**
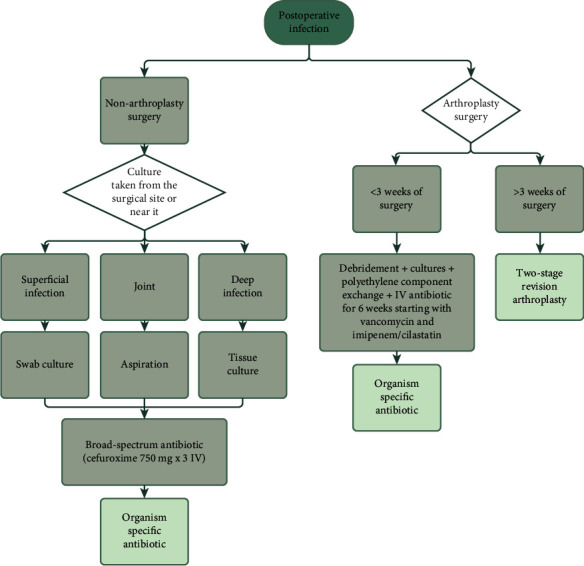
The treatment algorithm used in JUH for patients with postoperative orthopedic infection.

Gram-negative bacteria are more resistant to antibiotics in comparison to gram-positive [30, 31]. These pathogens have developed a variety of mechanisms to counteract the action of antimicrobials, such as *β*-lactamase production, minimizing antimicrobial agent penetration, target site alterations, and efflux pumps [32]. The results of this analysis demonstrated that *Klebsiella* species showed high resistance against co-trimoxazole (80%), cefazolin (100%), and ampicillin (100%). These findings are comparable to those of a study investigating the surgical site infections at a tertiary hospital in India, where drug resistance was 93.6% and 95.7% to cefazolin and ampicillin, respectively [33]. These findings indicate that those antibiotics are unusable, leaving physicians with limited options. Carbapenems are considered the most powerful class of *β*-lactams; therefore, these drugs are reserved for treating severe infections in hospital settings [33]. However, our results showed that Acinetobacter species had high resistance to ertapenem (75%), imipenem (80%), and meropenem (100). Our findings go hand in hand with what Tuon et al. found in their study, where meropenem had a resistance rate of 100% [10].  Figure 2 shows the treatment algorithm used in JUH for patients with postoperative orthopedic infection.

The financial impact of surgical site infections is a worldwide challenge for the healthcare system [34–36]. In the United States, it is considered the third most expensive healthcare-acquired infection to treat with an estimation of 20785 US dollars [37]. A study in Jordan showed that the mean healthcare costs of patients with surgical site infections are approximately twice as high as that of noninfected patients [38]. It is worth noting that these costs are affected by a variety of factors, including the severity of the infections, characteristics of the patients and comparators, hospital settings, and the type of medical costs [36]. In addition to the financial burden, surgical site infections in orthopedic patients impact patients' quality of life and limit their physical activities [39].

One of the strengths of this study is that it represents a comprehensive assessment of the characteristics of postoperative infections in orthopedic practice at Jordan University Hospital, including their antimicrobial susceptibility pattern. This is particularly important due to the fact that there is no regulating body concerned with the appropriate dispensing of antibiotics in Jordan. However, this study has some limitations. Firstly, because our study was carried out in a retrospective design, we had to rely solely on the patients' records to confirm the presence or absence of infections; therefore, misclassification bias and underreporting of cases could not be ruled out. Secondly, we collected only the records of the infected patients without taking into account other patients who underwent operations, preventing us from comparing the two groups.

## 5. Conclusions

Our results showed that the postoperative infection rate at JUH is lower than that of other developing country hospitals. Furthermore, *S*. *aureus* species were the most isolated bacteria, with a concerningly high proportion being MRSA. This emphasizes the need to strictly regulate antibiotic dispensing through the collaboration of the authorities, physicians, and pharmacists, as well as, supporting the initiatives that call for smart use and stewardship of antibiotics which have been shown to be effective in Jordan [40].

## Figures and Tables

**Table 1:** Characteristics of cases with postoperative infection.

Characteristics	*n* (%)
*Age (years)*	
≤18	32 (20.3)
19–40	48 (30.4)
41–60	28 (17.7)
>61	50 (31.6)

*Gender*	
Male	100 (63.3)
Female	58 (36.7)

*Location of surgery*	
JUH	110 (69.6)
Outside JUH	48 (30.4)

*Type of surgery*	
Trauma	84 (53.2)
Elective	69 (43.7)
Emergency	5 (3.2)

*Comorbidities* ^a^	
Hypertension	42 (26.6)
Diabetes mellitus	38 (24.1)
Ischemic heart disease	11 (7)
Chronic kidney disease	7 (4.4)
Thyroid disease	3 (1.9)
None	103 (65.2)

JUH, Jordan University Hospital; the symbol ^a^ indicates that the sum is greater than 100% because some patients had more than one disease.

**Table 2:** Frequency of bacteria in infected patients.

Microorganisms	*n* (%)	Age (years ± SD)	Sex (male %)	Postoperative infection (JUH) *n* (%)	Postoperative infection (referred) *n* (%)
*Gram-positive*					
*Staphylococcus aureus*	64 (38.1)	35.5 ± 23.6	60.9	48 (28.6)	16 (9.5)
Coagulase-negative staphylococci	40 (23.8)	51.7 ± 23.6	70	21 (12.5)	19 (11.3)
*Enterococcus* species	10 (6)	59.7 ± 22.7	80	7 (4.2)	3 (1.8)
*Staphylococcus* species	1 (0.6)	80.0 ± 0.0	100	1 (0.6)	0
Diphtheroid bacilli	1 (0.6)	73.0 ± 0.0	0	0	1 (0.6)
*Bacillus* species	1 (0.6)	70.0 ± 0.0	0	0	1 (0.6)
Actinomyces odontolyticus	1 (0.6)	14.0 ± 0.0	100	0	1 (0.6)

*Gram-negative*					
*Klebsiella* species	14 (8.3)	49.6 ± 23.1	64.3	12 (7.2)	2 (1.2)
*Escherichia coli*	10 (6)	64.7 ± 19.1	40	7 (4.2)	3 (1.8)
*Pseudomonas aeruginosa*	10 (6)	30.6 ± 20.7	70	7 (4.2)	3 (1.8)
*Enterobacter* species	5 (3)	45.0 ± 22.0	60	4 (2.4)	1 (0.6)
Acinetobacter species	5 (3)	54.8 ± 26.8	60	3 (1.8)	2 (1.2)
Alcaligenes species	2 (1.2)	25.0 ± 0.0	0	2 (1.2)	0
Citrobacter species	2 (1.2)	45.0 ± 22.6	100	1 (0.6)	1 (0.6)
Burkholderia species	1 (0.6)	25.0 ± 0.0	100	1 (0.6)	0
Comamonas testosteroni	1 (0.6)	64.0 ± 0.0	0	0	1 (0.6)

JUH, Jordan University Hospital; data were calculated based on 168 identified bacteria (in 9 cases more than one pathogen was found).

**Table 3:** Resistance pattern of gram-positive bacteria to antibiotic agents.

Antibiotics	*Staphylococcus aureus*	CoNS	*Enterococcus* spp.	Diphtheroid bacilli	*Bacillus* spp.
	*n* = 64	*n* = 40	*n* = 10	*n* = 1	*n* = 1
Ampicillin	36 (91.7)	28 (85.7)	8 (37.5)	1 (0)	1 (0)
Levofloxacin	61 (9.8)	40 (45)	8 (75)	1 (100)	1 (0)
Gentamicin	61 (8.2)	39 (33.3)	7 (71.4)	1 (0)	1 (0)
Ciprofloxacin	32 (12.5)	11 (45.5)	4 (100)	—	—
Vancomycin	62 (1.6)	40 (0)	8 (0)	1 (0)	1 (0)
Oxacillin	62 (77.4)	40 (87.5)	—	1 (100)	1 (100)
Erythromycin	63 (41.3)	40 (55)	8 (75)	1 (100)	1 (100)
Clindamycin	62 (29)	40 (30)	1 (100)	1 (100)	1 (100)
Linezolid	26 (0)	11 (0)	5 (20)	—	—
Tigecycline	53 (1.9)	36 (8.3)	6 (16.7)	1 (0)	1 (0)
Co-trimoxazole	31 (25.8)	11 (18.2)	1 (0)	—	—
Nitrofurantoin	19 (0)	8 (0)	4 (0)	—	—
Benzylpenicillin	32 (96.9)	11 (100)	4 (50)	—	—
Moxifloxacin	30 (6.7)	11 (18.2)	1 (100)	—	—
Tetracycline	31 (25.8)	10 (20)	4 (100)	—	—
Chloramphenicol	34 (2.9)	30 (13.3)	7 (14.3)	1 (0)	1 (100)
Quinupristin	9 (0)	1 (0)	1 (100)	—	—
Amoxicillin/clavulanic acid	2 (100)	—	1 (0)	—	—

Data are represented in *n* (%); data were calculated based on 168 identified bacteria (in 9 cases more than one pathogen was found); CoNS, coagulase-negative *Staphylococci*; spp., species.

**Table 4:** Resistance pattern of gram-negative bacteria to antibiotic agents.

Antibiotics	*Klebsiella* spp.	*Escherichia coli*	*Pseudomonas aeruginosa*	*Enterobacter* spp.	*Acinetobacter* spp.
	*n* = 14	*n* = 10	*n* = 10	*n* = 5	*n* = 5
Ampicillin	5 (100)	4 (100)	—	—	—
Levofloxacin	5 (60)	4 (25)	1 (100)	3 (66.7)	1 (100)
Gentamicin	14 (50)	10 (30)	9 (0)	5 (20)	5 (60)
Ceftriaxone	14 (64.3)	10 (50)	—	5 (60)	5 (60)
Cefoxitin	9 (44.4)	6 (66.7)	—	3 (100)	—
Amikacin	14 (21.4)	10 (0)	10 (20)	5 (0)	4 (50)
Ciprofloxacin	14 (42.9)	10 (60)	10 (20)	5 (40)	4 (75)
Cefazolin	4 (100)	4 (50)	—	3 (100)	1 (100)
Imipenem	14 (35.7)	10 (0)	10 (30)	5 (0)	5 (80)
Ertapenem	14 (35.7)	10 (0)	—	5 (20)	4 (75)
Ceftazidime	14 (64.3)	10 (50)	10 (30)	5 (60)	5 (100)
Tigecycline	13 (15.4)	8 (0)	1 (0)	4 (0)	5 (40)
Cefepime	13 (53.8)	9 (66.7)	10 (70)	5 (40)	5 (100)
Cefotaxime	9 (44.4)	6 (33.3)	—	2 (50)	4 (75)
Co-trimoxazole	5 (80)	4 (75)	2 (100)	3 (33.3)	1 (0)
Nitrofurantoin	3 (66.7)	1 (0)	—	1 (100)	—
Meropenem	1 (0)	—	9 (55.6)	1 (0)	4 (100)
Piperacillin/tazobactam	5 (60)	2 (0)	9 (33.3)	3 (0)	4 (100)
Colistin sulfate	1 (0)	—	2 (0)	—	4 (0)
Minocycline	—	—	2 (100)	—	1 (0)
Piperacillin	—	—	4 (50)	—	1 (100)
Ticarcillin/clavulanic acid	—	—	4 (50)	—	1 (100)
Tobramycin	—	—	4 (0)	—	1 (0)
Ticarcillin	—	—	3 (33.3)	—	1 (100)
Aztreonam	1 (100)	—	6 (50)	1 (0)	3 (66.7)
Cefoxitin	5 (60)	4 (50)	1 (100)	2 (100)	4 (100)

Data are represented in *n* (%); data were calculated based on 168 identified bacteria (in 9 cases more than one pathogen was found); spp., species.

**Table 5:** Comparison between the resistance pattern according to the source of infection for the most common bacteria.

Antibiotics	*Staphylococcus aureus*		CoNS		*Enterococcus* species	
	JUH	Referred	JUH	Referred	JUH	Referred
Ampicillin	23 (87)	13 (100)	14 (100)	14 (71.4)	5 (20)	3 (66.7)
Levofloxacin	45 (6.7)	16 (18.8)	21 (38.1)	19 (52.6)	5 (80)	3 (66.7)
Gentamicin	46 (6.5)	15 (13.3)	20 (35)	19 (31.6)	5 (80)	2 (50)
Ciprofloxacin	26 (7.7)	6 (33.3)	4 (25)	7 (57.1)	3 (100)	1 (100)
Vancomycin	46 (0)	16 (6.3)	21 (0)	19 (0)	6 (0)	2 (0)
Oxacillin	47 (74.5)	15 (86.7)	21 (100)	19 (73.7)	—	—
Erythromycin	47 (34)	16 (62.5)	21 (71.4)	19 (36.8)	5 (100)	3 (33.3)
Clindamycin	47 (25.5)	15 (40)	21 (28.6)	19 (31.6)	1 (100)	—
Linezolid	21 (0)	5 (0)	4 (0)	7 (0)	3 (0)	2 (50)
Tigecycline	39 (2.6)	14 (0)	17 (17.6)	19 (0)	6 (16.7)	—
Co-trimoxazole	26 (23.1)	5 (40)	4 (0)	7 (28.6)	1 (0)	—
Nitrofurantoin	16 (0)	3 (0)	2 (0)	6 (0)	3 (0)	1 (0)
Benzylpenicillin	26 (96.2)	6 (100)	4 (100)	7 (100)	3 (66.7)	1 (0)
Moxifloxacin	25 (8)	5 (0)	4 (0)	7 (28.6)	1 (100)	—
Tetracycline	25 (24)	6 (33.3)	4 (0)	6 (33.3)	3 (100)	1 (100)
Chloramphenicol	22 (4.5)	12 (0)	16 (18.8)	14 (7.1)	4 (0)	3 (33.3)
Rifampin	1 (0)	—	—	—	—	—
Quinupristin	7 (0)	2 (0)	1 (0)	—	1 (100)	—

Data are represented in *n* (%); data were calculated based on 168 identified bacteria (in 9 cases more than one pathogen was found); CoNS, coagulase-negative *Staphylococci*; JUH, Jordan University Hospital.

## Data Availability

The data from the present research that were utilized and analyzed are accessible from the corresponding author upon reasonable request.
